# A local vascularized scapula bone graft to treat posterior glenohumeral instability—an innovative surgical technique

**DOI:** 10.1016/j.xrrt.2026.100772

**Published:** 2026-05-05

**Authors:** Philipp Lamby, Norbert Heine, Andreas Eigenberger, Christoph Eckstein, Lukas Prantl, Johannes Zellner, Paul Schmitz

**Affiliations:** a Department of Plastic and Reconstructive Surgery, University Medical Centre Regensburg, Regensburg, Germany; b Department of Trauma Surgery, University Medical Centre Regensburg, Regensburg, Germany

**Keywords:** Posterior glenohumeral instability, PGHI, Posterior shoulder instability, Bone allograft, Bone transplant, Vascularized bone graft

## Abstract

**Background:**

Posterior glenohumeral instability (PGHI) is a relatively rare functional disability. In cases of significant bony or soft tissue defects, surgical treatment is often unavoidable. Based on the type of defect, a soft tissue procedure, a glenoid reconstruction/augmentation with an allograft, or a combined surgical procedure must be performed. Procedures using avascular allografts from different harvest areas or fresh cadaveric specimens are well established. Nevertheless, in approximately 20% of cases, resorption or aseptic necrosis of the graft leads to recurrent symptoms and repeated glenohumeral dislocation. To overcome this serious disadvantage, vascularized bone grafts should be used.

**Methods:**

We introduce an innovative surgical technique using a locally pediculated vascularized bone graft from the scapular pillar to treat PGHI associated with hereditary hypotrophy of the glenoid.

**Results:**

The two-year follow-up of the performed surgical procedure led to a posteriorly stable glenohumeral joint and a persisting vital bone graft. It is an innovative technique supplementing the broad spectrum of surgical techniques to treat PGHI.

**Conclusion:**

Treatment options for PGHI should be selected for each patient individually, considering the causes of instability as well as performing a thorough analysis of the bone stock.

Posterior glenohumeral instability (PGHI) is a relatively rare functional disability caused by a variety of injuries, muscular imbalance, dysmorphic shoulder anatomy, and congenital disorders.^9^ Especially in young male contact athletes, it is recognized in up to 10% of all instability events.^27^^,^^30^^,^^39^^,^^50^ The most common form of PGHI is recurrent posterior subluxation, which is often noticed by the patient due to pain and/or weakness rather than a full luxation or subluxation of the glenohumeral joint.^6^^,^^30^

In the majority of cases, conservative treatment is effective if no critical bony or soft tissue defect is present.^49^ Nonetheless, numerous surgical procedures have been developed over time to treat chronic or recurrent PGHI. These procedures can be divided into 3 main categories:

1 Soft tissue–based procedures
2 Bone-based procedures
3 Combined procedures (bone- and soft tissue–based) (Table I).

**Table I:** Established surgical procedures to treat PGHI.

Procedure	Aim	Surgical technique	References
Soft tissue based			
Capsular shift	Reduce capsular and ligamentous redundancy	Superior shift of the posterior-inferior capsular	Neer–Foster^36^ Bigliani^2^ Fuchs^14^
Labral repair	Fix a labral detachment (reverse Bankart lesion) to the posterior glenoid	Open or arthroscopic labral repair using bioabsorbable suture anchors **(Remark:** very successful in combination with a capsulorrhaphy or capsular plication)	Hawkins-Janda^18^ Kim^25^ Bottoni^3^ DeLong^9^
Bone based			
Glenoid bone block 1. Extracapsular	Increase the posterior glenoid surface in case of recurrent posterior luxation or failed previous procedures	**a.** iliac bone block from the ipsilateral iliac crest (**Remark**: resorption of the graft in 20%^13^) **b.** acromial bone block with a pediculated deltoid flap	Hindenach^20^ Mowery^35^ Barbier^1^ Sirveaux^46^
Glenoid bone block 2. Intracapsular	Provide an anatomic reconstruction of the glenoid cavity in recurrent PSI combined with glenoid bone loss exceeding more than 25% of the width of the inferior glenoid	Distal tibial allograft form fresh cadaveric specimens (**Remark**: long-term results are outstanding)	Millett^34^ Frank^12^
Glenoid osteotomy	Treat atraumatic posterior instability	**Remark:** technically demanding; high complication rates^4^^,^^15^^,^^41^	Scott^44^ Graichen^17^
Humeral osteotomy	Treat chronic or locked posterior dislocation by restoring glenohumeral congruity	Proximal derotational osteotomy of the humeral head **Remark:** rather uncommon; controversy discussed due to a high complication rate^5^^,^^43^^,^^48^	Vukov^47^ Keppler^24^ Ziran Nourbakhsh^54^
Combined			
Modified McLaughlin procedure	Treat an anterior humeral head bone defect (reverse Hill-Sachs/McLaughlin lesion)	Transfer of the subscapularis tendon from the lesser humeral tuberosity into the bony defect or transfer of the subscapularis and lesser tuberosity into the defect	McLaughlin^32^ Hawkins^19^

*PGHI*, posterior glenohumeral instability.

For the primary surgical treatment of a PGHI, arthroscopic soft tissue–based procedures are now very successful.^9^^,^^29^, ^30^, ^31^^,^^40^^,^^41^ In cases with recurrent PGHI, bony defects, or severe morphological abnormalities of the glenoid cavity, bone-based or combined procedures should be considered to achieve long-lasting posterior stability. On the humeral side, a large bony defect, known as a “reverse Hill–Sachs” or “McLaughlin” lesion, requires a reconstruction of the “glenoid track,” which represents a key factor in maintaining joint stability.^22^^,^^37^ Concerning the glenoid cavity, structural variables, post-traumatic bony defects, or progressive eccentric posterior glenoid wear can sustainably influence shoulder stability if not reconstructed. Whereas glenoid or humeral osteotomies are powerful but technically demanding procedures with a high complication rate, extracapsular and intracapsular bone grafts are performed routinely to increase the posterior glenoid surface or even provide an anatomical reconstruction of the glenoid cavity.

Unfortunately, a disadvantage of avascular bone grafts is the resorption/aseptic necrosis of the graft in about 20% of cases, leading to recurrent symptoms for the patient and recurrent glenohumeral dislocation.^13^^,^^16^

To overcome this serious complication, we introduce an innovative surgical technique for stabilization of chronic PGHI using a locally vascularized bone graft (VBG) from the ipsilateral scapula pillar. The short distance between the glenoid and the lateral border of the scapula allows a local transposition of the bone graft on its nourishing vascular pedicle arising from the circumflex scapular artery.

The lateral border of the scapula has never been reported to be suitable for glenoid augmentation. Thus, we first had to prove the concept with regard to the size match of the scapula pillar and the glenoid in a 3-dimensional (3D)-printed model.

## Patient medical history

**Figure 1:**
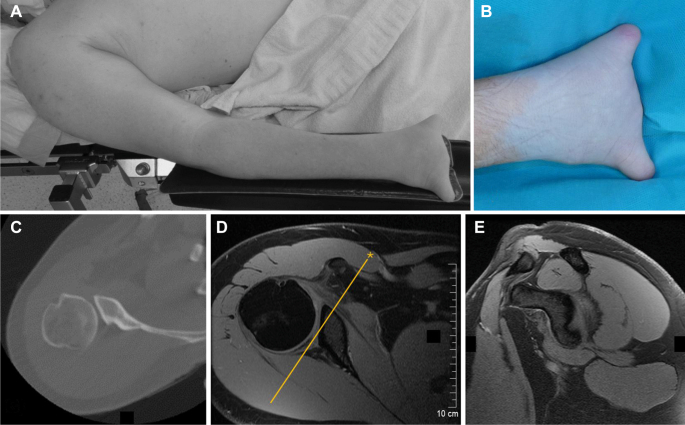
(**A** and **B**) Patient exhibited dysmelia with incomplete stump formation but symmetrical shoulder girdle and upper arm musculature. (**C**) Pre-operative axial CT image showing a posterior glenohumeral instability (Walch type C). (**D** and **E**) MRI prior to the first surgical glenoid reconstruction. *CT*, computer tomography; *MRI*, magnetic resonance imaging.

We report on a male patient who was first seen at our clinic at the age of 26 for PGHI. The PGHI was caused by a hereditary hypotrophy of the posterior part of the glenoid (Fig. 1). In the same upper extremity, the patient exhibited dysmelia with incomplete stump formation (Fig. 1). The patient's shoulder girdle and upper arm musculature were symmetrical, and he was able to use the affected limb normally in both his personal and professional life. As a secondary finding, the patient had medically controlled hypothyroidism. The patient was not aware of any syndromic clinical picture. Based on a review of the available clinical findings (hypothyroidism, dysmelia of the upper extremities, and hereditary glenoid hypotrophy), we were unable to identify a syndromic clinical picture. A stabilization using an avascularized iliac crest bone graft led to primary stability but ultimately resulted in recurrent instability after 6 months due to partial resorption of the graft, following which the patient presented at our clinic.

## Three-dimensional planning and surgical technique

**Figure 2:**
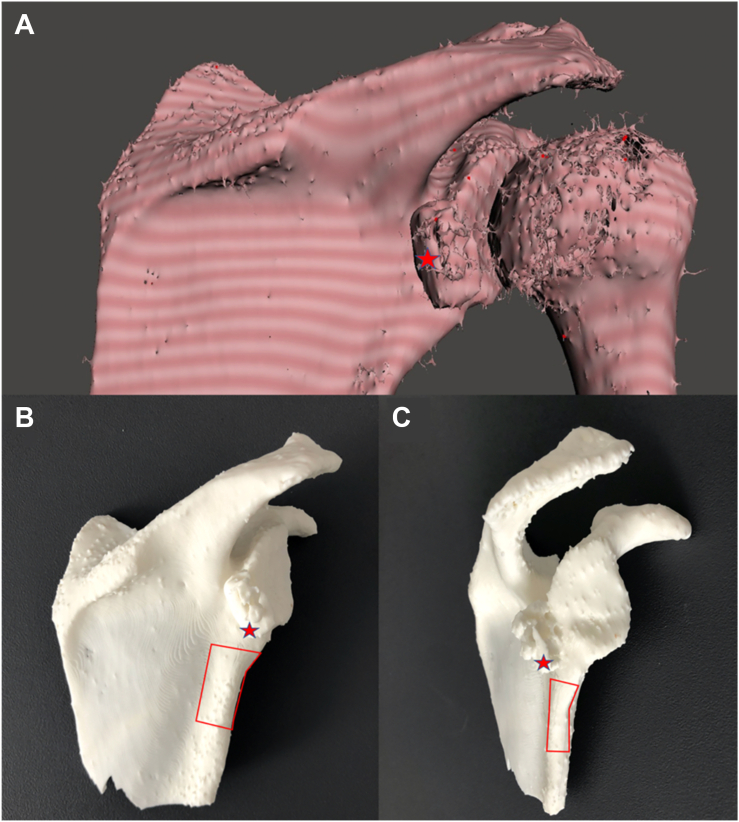
PGHI with avascular iliac crest bone graft marked with asterisk. (**A**) 3D model of the pre-operative scenario. (**B** and **C**) 3D print of the pre-operative scenario. Red polygon marks the planned vascularized bone graft within the lateral border of the scapula. *PGHI*, posterior glenohumeral instability; *3D*, three-dimensional.

For surgical planning, an optimal visualization of the scapula was required. Therefore, a 3D model was created from pre-operative tomographic images of the shoulder region using InVesalius 3.1 software (CTI, Campinas, São Paulo, Brazil) (Fig. 2). Using Autodesk Meshmixer (San Rafael, CA, USA), the scapula was isolated, artifacts were deleted, and the surface was closed. Several times, the scapula was printed at original size in white polylactic acid using the Original Prusa Mini+ (Prusa Research, Prague, Czech Republic). For visualization of the surgical outcome, these steps were also carried out for the 3-month post-operative scan.

## Surgical technique

### Patient positioning

The surgery is performed under general anesthesia. The patient is positioned in the lateral decubitus position with the operative side facing up. The patient is secured by padded side supports. The operated arm is positioned in 90° flexion over a bolster while being able to be fully mobilized. The contralateral side is cushioned to prevent compression of the axilla. Preferably, a vacuum mattress is used to increase stability. The sterile drapes are standard for scapula flap procedures.

### Harvesting step

Initially, the location of the desired bone flap is marked pre-operatively. The incision is made along the lateral border of the scapula. Subcutaneous tissue and fascia will be dissected with care to ensure the safety of the circumflex scapular artery. Afterward, after identifying the circumflex scapular artery within the lateral axillary foramen, it will be dissected until a pedicle with a length of 6-9 cm has been harvested.

Next, the teres minor and the long head of the triceps are mobilized from the lateral border of the scapula. The bone can be harvested approximately 2 cm below the glenohumeral joint, with a width of 1-2 cm and a length of 4 cm. The exact dimensions can vary from patient to patient. The bone will remain pedicled to the circumflex scapular artery to ensure blood supply. The periosteum is preserved to maintain vascularity. After harvesting the bone, the mobilized muscle will be reinserted into the scapula again.

### Positioning step

After the harvesting of the bone flap, it is carefully rotated and positioned on the posterior glenoid rim to restore posterior bony support. The bone flap is initially fixed in the desired position by 2 K-wires. After confirming the correct position using intraoperative x-ray imaging, the flap is fixed with 2 cannulated 4.2 mm titanium cortex screws. Post-operative care includes 6 weeks of immobilization to ensure bone integration, as well as rehabilitation, initially passive, which is then followed by active mobilization. To illustrate the surgical effect, a pre-operative and a post-operative CT scan are shown in the section Patient medical history and Results.

## Results

**Figure 3:**
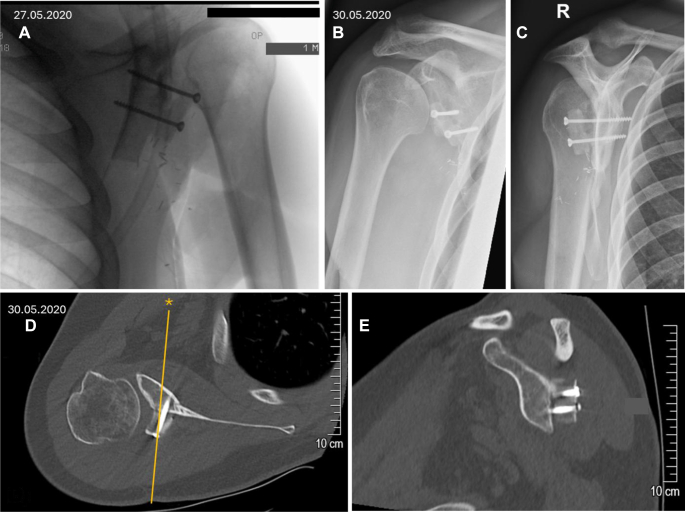
(**A**) Intraoperative fluoroscopy of the vascularized scapular bone flap and screw fixation. (**B** and **C**) Post-operative x-ray of the right shoulder after vascularized scapular bone flap and screw fixation. (**D** and **E**) Post-operative axial CT image demonstrating successful placement of the scapular bone flap. *CT*, computer tomography.

In the current clinical case, the surgical technique for the local vascularized scapula bone graft was performed as described above. Distinct technical challenges were encountered as a result of the previous surgical intervention. Since partial resorption of an iliac crest graft was already present, the necrotic tissue and the screws inserted during the prior operation had to be removed intraoperatively. The already limited exposure of the shoulder joint was further restricted by post-operative adhesions. The recipient site was débrided with a Luer so that vital cancellous bone formed the bed for the VBG. In addition, the considerable muscular bulk surrounding the joint, particularly the rotator cuff complex, substantially reduced the available space for precise graft placement. Intraoperative visualization through a dorsal approach provided only limited orientation. The described technique may be complemented by a simultaneous shoulder arthroscopy, which allows comprehensive joint inspection, identification, and immediate management of concomitant intra-articular lesions. Figure 3 shows the intraoperative fluoroscopy following the transplantation of the local vascularized scapula bone graft; Figure 3 also depicts the post-operative conventional X-rays and the CT images demonstrating successful placement of the scapular bone flap as well as the resulting dorsal support.

**Figure 4:**
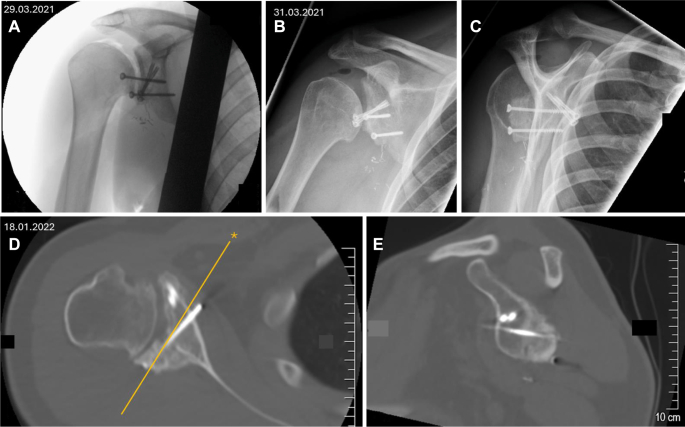
(**A**) Intraoperative fluoroscopy of the coracoid bone transposition in the Latarjet procedure to treat ventral shoulder dislocation. (**B** and **C**) Post-operative x-ray of the right shoulder two days after coracoid bone transposition and one year after posterior vascularized scapular bone flap. (**D** and **E**) 2-year follow-up: axial CT image demonstrating vital posterior and anterior bone flaps. *CT*, computer tomography.

However, despite an initially satisfactory healing process, increasing strength in the affected shoulder, and improved range of motion, the patient exhibited an anterior shoulder instability 6 months after surgery. This condition could not be resolved through conservative treatment, including intensive physical therapy over a 6-month period. Consequently, an indication for anterior shoulder stabilization surgery via the Latarjet procedure was established, and the procedure was performed (Fig. 4).

We observed a continued satisfactory course of recovery up to 2 years after the primary surgery. In the final clinical and computed tomography examinations, a firm, stable, and vitally healed vascularized scapular bone graft was also detected (Fig. 4).

## Discussion

The current gold standard for bone-based glenoid reconstruction to treat PGHI is the transplantation of a nonvascularized bone graft (NVBG). Usually, an NVBG from the ipsilateral iliac crest is harvested and transplanted to the posterior part of the scapula to increase or reconstruct the surface of the glenoid cavity. After débridement of the insertion area, the bony transplant is fixed with 2 lag screws. In the majority of uncomplicated patients, this surgical procedure, in combination with regular, specific physical exercise, leads to a stable joint. Unfortunately, resorption of the graft occurs in up to 20%, consecutively leading to recurrent instability of the glenohumeral joint.^13^

In anterior glenohumeral instability and bone loss, a VBG using part of the ipsilateral coracoid bone was introduced, known as the Latarjet procedure.^28^ This technique maintains blood supply for the bone graft through the conjoined tendon. Even though graft resorption of the vascularized coracoid bone has been reported to be as low as 5-20%, VBGs are clearly superior to non-VBGs concerning this matter (*P* < .001).^7^^,^^8^

An alternative treatment to autologous NVBG is a fresh cadaveric distal tibia allograft (AlloSource, Denver, CO, USA). The lateral third of the distal tibial platform is prepared to match the bony defect of the posterior glenoid. The allograft is fixed to the scapula by 2 screws, and thus an anatomical reconstruction of the glenoid surface is achieved, as postulated by Barbier et al.^1^ In comparison to a fresh cadaveric specimen, a benefit of vital bone grafts is that they provide osteoconductive, osteoinductive, and osteogenic properties. These properties, and the feasibility of graft growth, can even be enhanced using VBGs. Furthermore, VBGs show a high rate of successful graft incorporation.^11^^,^^51^ Especially in large bony defects, VBGs have shown their superiority, maintaining radiologic density 1.5 to 2 times better than NVBGs. Besides the rapid incorporation, VBGs maintain long-term stability under compressive and rotary forces. Furthermore, they sustain the ability to treat active infections in situ.^42^^,^^52^

For the treatment of PGHI, a surgical technique using an acromial bone block with a pedicled deltoid flap was described by Kouvalchouk, enabling triple shoulder locking through the blocking effect, the retention hammock provided by the deltoid flap, and posterior capsule repair.^46^ A derivative arthroscopic-assisted technique using an acromial pedicled bone block was described by Métais et al.^33^

According to the required size and shape of the bone graft, as well as the need for a weight-bearing or non-weight-bearing bone graft, different regions of the body can provide VBGs. Furthermore, the region for reconstruction and the comorbidities that accompany harvesting determine the area for bone harvesting. In general, VBGs from the distal radius, vascularized portions of the metatarsals can be used for small bone defects, a vascularized rib and a vascularized medial femoral condyle, as well as vascularized scapula and vascularized posterior iliac crest bone grafts, can be used for an average bony defect, whereas the vascularized fibula remains the gold standard for reconstruction of long bony defects.^21^

Pre-operative analysis and matching the bone graft to the recipient area are crucial to create the basis for surgical success. Recently, virtual planning and 3D printing templates have become increasingly popular to prepare for surgical treatment, especially in craniofacial reconstruction and hand surgery, but also in orthopedic surgery.^6^^,^^10^^,^^45^^,^^53^ Innovative techniques, such as creating a 3D printed model, provide the opportunity to visualize the shape of the glenoid and focus on the pre-operative scenario. Even the microstructure of the bone graft, specifically the relative bone volume, surface density, trabecular thicknesses, and trabecular separation, can be analyzed pre-operatively using high-resolution datasets generated by micro-computed tomography.^23^ Creating 3D models of the anatomy and the bone graft recipient area allows easy interpretation and planning by surgeons.^10^^,^^45^^,^^53^ Furthermore, it can shorten the operative time as well as improve the post-operative outcome.^38^

Taking the abovementioned points into account, we decided to establish a new surgical technique as a salvage procedure using a local VBG to treat a failed NVBG for the reconstruction of a hereditary hypotrophy of the posterior part of the glenoid. Especially the success of the Latarjet procedure and its superiority compared to non-VBGs convinced us to establish a procedure that uses a vascularized graft.^8^ In addition, we believe that the surgical technique we described is even superior to the Latarjet procedure in terms of blood supply, since the blood supply is provided by a single, macroscopically visible arteriovenous bundle, whereas in the Latarjet procedure, the blood supply is maintained via microvascular vessels accompanying the tendon.

Even though the described technique is very promising to overcome the fateful disadvantage of aseptic necrosis of an NVBG, the described procedure has certain limitations. First of all, the procedure certainly needs a significant additional operative time in comparison to harvesting and transplanting an NVBG. Secondly, the used local VBG from the ipsilateral scapula pillar fails to provide anatomic restoration of the glenoid articular surface, just like most bone block-based glenohumeral reconstruction procedures. Thus, early symptomatic glenohumeral arthritis is to be expected. Nevertheless, a vital, stable bone stock is indispensable for durable stability of the glenohumeral joint as well as for subsequent therapies.

The described procedure can be amended by a simultaneous shoulder arthroscopy, allowing joint exploration, detection, and immediate treatment of associated intra-articular lesions. Furthermore, an intraoperative arthroscopic control visualizes the achieved stability of the humeral-glenoid joint. An advantage of arthroscopically assisted bone grafting for anterior shoulder stabilization was demonstrated by Kordasiewicz et al regarding less nonunion and development of osteolysis/resorption of the bone graft.^26^

A minor benefit of the described procedure is that the harvesting of the VBG and the stabilizing reconstruction of the PGHI are performed via one approach, and no additional incision is required. Furthermore, the blood supply to the graft is always maintained throughout the whole surgical intervention, in contrast to a free-transfer VBG or an NVBG.

The 2-year follow-up of the patient showed a persisting vital bone graft and a posteriorly stable glenohumeral joint. In the CT follow-up examinations, the VBG appeared to be firmly and stably anchored. No resorption of the bone graft was observed (Fig. 4). Long-term outcomes of the proposed surgical procedure remain unknown. Nevertheless, this innovative technique adds to the broad spectrum of surgical techniques to treat PGHI and to achieve a stable articular function, including a full range of motion with no or reduced complaints from the patient.

## Conclusion

Posterior shoulder instability is caused by various rare congenital diseases in combination with muscular imbalance and/or a dysmorphic glenoid, as well as by different types of injuries.^9^ Current treatment strategies for PGHI favor lesion-specific techniques. In cases of bony defects or a dysmorphic glenoid, bone allografts are routinely used to reconstruct or augment the glenoid.^9^^,^^13^^,^^16^^,^^29^, ^30^, ^31^^,^^40^^,^^41^ Even though these surgical techniques are well established and commonly performed, a high rate of post-operative bone graft necrosis occurs, leading to recurrent instability.^13^^,^^16^ This unintended complication can be overcome with the surgical procedure of a locally vascularized scapular bone graft, described in this publication for the first time. Nevertheless, PGHI remains a challenging condition, requiring restoration of posterior capsulolabral structures and glenoid bone stock. Therefore, the best treatment option should be selected for each patient, considering the individual causes of instability as well as the patient's expectations and needs.

## Data availability statement

No new data were created or analyzed in this study. Data sharing is not applicable to this article.

## Disclaimers:

Funding: No funding was disclosed by the authors.

Conflicts of interest: The authors, their immediate families, and any research foundations with which they are affiliated have not received any financial payments or other benefits from any commercial entity related to the subject of this article.
